# Structure/function studies of dogfish α-crystallin, comparison with bovine α-crystallin

**Published:** 2009-11-20

**Authors:** A. Ghahghaei, A. Rekas, J.A. Carver, R.C. Augusteyn

**Affiliations:** 1 Biology Department, University of Sistan and Baluchestan, Zahedan, Iran; 2 National Deuteration Facility, Australian Nuclear Science and Technology Organization, Lucas Heights, New South Wales, Australia; 3 School of Chemistry & Physics, The University of Adelaide, South Australia, Australia; 4 Biochemistry Department, La Trobe University, Victoria, Australia

## Abstract

**Purpose:**

α-Crystallin is the major protein of the mammalian lens where it contributes to the refractive properties needed for vision and possibly to the stability of the tissue. The aim of this study was to determine whether the properties of α-crystallin have changed during the course of evolution.

**Methods:**

Dogfish α-crystallin, which appeared over 420 million years ago, has been contrasted with bovine α-crystallin, which emerged around 160 million years later, by comparing their sizes, the microenvironments of their cysteine and tryptophan residues, their chaperone-like activities and the flexibility of their COOH-terminal extensions.

**Results:**

Dogfish α-crystallin consists of αA- and αB-polypeptides, in a 1:5 ratio, and has a molecular mass of around 400 kDa. By contrast, the bovine protein is around 600-800 kDa in mass and has a 3:1 subunit ratio. Cysteine residues in the proteins were equally accessible to reaction with 5,5'-dithiobis-(2-nitrobenzoic acid). Quenching of fluorescence with acrylamide indicated tryptophan residues in the two proteins were in similar environments. The chaperone activity of dogfish α-crystallin was comparable to that of bovine α-crystallin in preventing the heat-induced precipitation of β_L_-crystallin but the dogfish protein was three times more effective at preventing insulin precipitation after reduction at 37 ˚C. ^1^H nuclear magnetic resonance spectroscopic studies showed that the last 17 amino acids of the dogfish αB polypeptide (V162-K178) have great conformational flexibility, are highly exposed to solvent and adopt little ordered conformation. This is comparable to, but slightly longer in length, than the COOH-terminal extension observed in mammalian α-crystallins.

**Conclusions:**

The structure and properties of α-crystallin have changed relatively little during the evolutionary period from the emergence of sharks and mammals.

## Introduction

α-Crystallin is the major protein component (up to 50%) of most eye lenses [1]. It is generally isolated as a polydisperse population of hetero-oligomers, with most averaging 600-800 kDa in mass for the young bovine protein and consisting of two polypeptides, αA- and αB-crystallin. These are present in molar ratios varying from 1:5 in the dogfish to 10:1 in the kangaroo [1-3] (unpublished observations). The sequences of the two polypeptides are closely related and have changed relatively little during evolution.

The α-crystallins belong to the small heat-shock protein (sHsp) family [4] and, under stress conditions, can act as molecular chaperones by preventing the precipitation of partially unfolded target proteins [5] via the formation of a stable, soluble, high molecular-mass sHsp-target protein complex [6]. Unlike other molecular chaperones that are involved in protein folding, α-crystallin has no ability to refold target proteins [6] although it has been reported that it can enhance the recovery of activity from denatured enzymes [7]. It has been proposed that the chaperone activity of α-crystallin, i.e. its ability to prevent crystallin protein aggregation, may be important in maintaining an optically transparent lens [5]. Thus, in zebrafish *cloche* mutants, over-expression of αA-crystallin prevents cataract formation arising from the insolubilization of γ-crystallins [8].

Most studies on the structure and properties of α-crystallin have been conducted with the human and bovine proteins. Comparisons of the mammalian proteins with evolutionary older proteins could yield valuable information on structure/function relationships in the sHsp family. The amino acid sequences of dogfish α-crystallin polypeptides display around 67% homology with those of the mammalian proteins [9]. It is therefore probable that they have homologous structural domains and comparable biological properties and functions. However, as with thermal stability [10], there may be differences in some properties necessary for the ectothermic dogfish proteins to exist at low and variable temperatures, very different conditions from their endothermic mammalian counterparts. Recent studies on other fish α-crystallins have revealed significant differences in the thermal stabilities of these proteins from endothermic and ectothermic species. The structure and function of the αA-crystallin appear to have been conserved but an αB-crystallin gene duplication in the zebrafish has led to a possible divergence of the structural and functional properties in the two polypeptides [11-14].

In order to examine whether the properties of α-crystallin may have changed during the course of evolution, the structures of dogfish and bovine α-crystallin have been compared by acquisition of ^1^H nuclear magnetic resonance (NMR) spectra and by assessing tryptophan and cysteine accessibilities. In addition, the ability of the proteins to act as molecular chaperones in suppressing the aggregation of stressed target proteins was compared. Our observations indicate that the dogfish and bovine proteins have similar structural properties and that there has been little change in α-crystallin since the divergence of the fish and mammalian lines.

## Methods

### Isolation of protein

Dogfish and bovine α-crystallins and bovine β-crystallin were prepared by gel filtration of lens extracts, at room temperature, on Sephacryl S300 in phosphate buffered saline containing sodium azide (PBSA) at pH 7.2, as described by Augusteyn et al. [15]. Only the central 50% of the α-crystallin peak was retained and, after concentrating, was rechromatographed under the same conditions. The process was repeated in order to remove any remaining traces of the adjacent high molecular weight (HMW) proteins and β-crystallin peaks. SDS PAGE was used to check that the preparations contained only α-crystallin polypeptides.

### Protein size analysis

The size of the proteins was examined by sedimentation velocity analysis as described by Thomson and Augusteyn [16] and by comparison with known proteins on gel permeation chromatography.

### Amino acid microenvironments

The reaction of cysteine residues with 5'-dithio-bis (2-nitrobenzoic acid; DTNB) under pseudo first order conditions was performed as described by Augusteyn et al. [17].

Accessibility of tryptophan to the solvent was examined by quenching of its fluorescence with acrylamide, as described previously [18].

### ^1^H NMR spectroscopy

Dogfish α-crystallin was concentrated by ultrafiltration and dialyzed against 11 mM phosphate buffer, pH 6.5. 0.033% NaN_3_. Buffer and D_2_O were then added to generate a solution containing 10% D_2_O and 20mg/ml protein in 10 mM phosphate buffer, 0.03% NaN_3,_ pH 6.5.

Two-dimensional ^1^H NMR experiments on dogfish α-crystallin were conducted at 500 MHz on a Varian Inova 500 NMR spectrometer (Varian Pty Ltd, Palo Alto, CA) at 25 °C. The sequential assignment procedure [19] of the ^1^H NMR resonances was undertaken using through-bond correlations via ^1^H-^1^H WATERGATE Total Correlation spectroscopy (TOCSY) [20] and through-space correlations via WET Nuclear Overhauser Effect Spectroscopy (NOESY) [21]. A spin-lock period of 65 ms was employed in the TOCSY experiment with dogfish α-crystallin as described by Carver et al. [22]. In TOCSY experiments, 32 scans were acquired for each of the 256 t_1_ increments. The free induction decay (FID) in t_2_ consisted of 4,096 data points over a spectral width of 6,000 Hz. A mixing time of 100 ms was used in the NOESY spectra with 80 scans for each increment. Other parameters were the same as those for the TOCSY experiments. Prior to the Fourier transformation, all spectra were processed using a shifted Gaussian function in both dimensions. The processing of NMR spectra was performed on a Sun Microsystems Sunblade 150 workstation (Sun Microsystems, San Francisco, CA) with Varian NMR software.

### Chaperone activity

The chaperone action of dogfish and bovine α-crystallins was assessed with two assays using different stresses, as described by Farahbakhsh et al. [23]. Assays were performed in duplicate.

#### 3rd-Insulin reduction assay

Insulin at 0.43 mg/ml in 50 mM sodium phosphate, pH 7.4, 0.05% (w/v) NaN_3_, was reduced with 20 mM dithiothreitol (DTT) in the presence of dogfish or bovine α-crystallin at various concentrations. The subsequent aggregation of the insulin B chain at 37 ^o^C was monitored as an increase in light scattered at 360 nm using a Spectramax 250 multiwell plate reader spectrophotometer (Molecular Devices, Sunnyvale, CA) with temperature control.

#### 3rd-Heat-stress assay

Bovine β_L_-crystallin at 0.3 mg/ml in 50 mM sodium phosphate pH 7.4, 0.05% (w/v) NaN_3,_ was incubated at 60 ^°^C in the presence of dogfish or bovine α-crystallin at various concentrations. The time-dependent aggregation of the protein was monitored by measuring light scattered at 360 nm using a Cary 500 Scan UV-VIS-NIR spectrophotometer (Varian Inc, Palo Alto, CA).

## Results

### Protein purification

**Figure 1:**
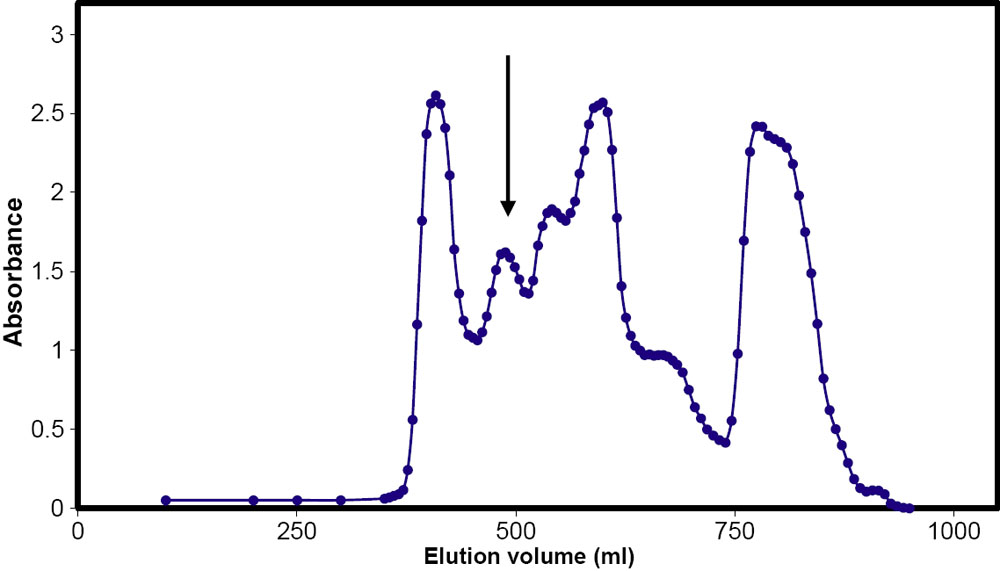
Fractionation of dogfish lens extract on a 3×150 cm Sephacryl S300 column. The position of the α-crystallin peak (490 ml) is indicated with an arrow. The central 50% of the peak was rechromatographed under the same conditions.

The size-exclusion elution profile for dogfish lens extract is shown in Figure 1. SDS-PAGE indicated that the peak eluting at 490 ml contained α-crystallin. It represented around 20%, by weight, of the soluble proteins. Use of Sephacryl S-300 column media, only utilizing the central portion of the α-crystallin peak, and further rechromatography of this portion, again taking only the central 50%, ensured that the isolated protein contained only α-crystallin. Under the same conditions, bovine α-crystallin eluted at around 450 ml, indicative of a smaller Stoke’s radius for the dogfish protein. By comparison with proteins of known size, it was estimated that the dogfish protein aggregate was around 400 kDa in mass and the bovine protein, approximately 600 kDa in mass. Sedimentation velocity analysis indicated that dogfish and bovine α-crystallins sedimented at 14.0 and 19.2 S, respectively. These data also imply that the dogfish protein aggregate is smaller in mass than the bovine protein. SDS-PAGE (not shown) revealed that the dogfish protein consisted of two subunits, αA- and αB-crystallin, in a ratio of approximately 1:5. This is similar to the observations of de Jong et al. [3].

### Amino acid microenvironments

**Figure 2:**
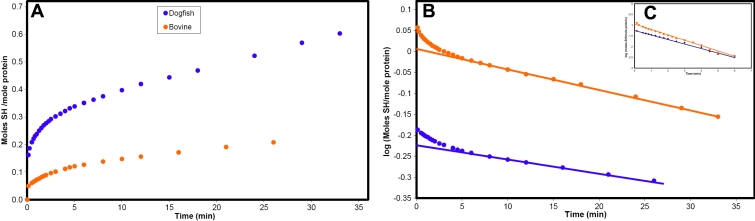
Thiol reactivity of a-crystallins. **A**: Time course for the reaction of dogfish and bovine α-crystallins with DTNB under pseudo first order conditions and **B**: first order log transform of the data in **A**, showing the lines of best fit for the slowly reacting (k=0.0034 and 0.0048) class of sulphydryls. **C**: First order log plot of the differences between the extrapolated and observed values in **B**, showing the more rapidly reacting (k=0.21 and 0.25) class of sulphydryls.

Analysis of the reaction of the α-crystallins with DTNB provided information on the accessibility of cysteine residues. The time courses for the reaction of bovine and dogfish proteins are presented in Figure 2A. The maximum SH content of the proteins, determined in the presence of 0.1% SDS, was 0.66 and 1.22 moles/mole for the bovine and dogfish proteins, respectively. First order transformation of these data (Figure 2B,C) indicated there were two classes of SH in each protein. In the bovine protein, 0.6 moles/mole subunit were in a slowly reacting, buried class (k=0.0034 min^-1^) and 0.06 in a more rapidly reacting, exposed class, (k=0.21 min^-1^) in agreement with a previous report [17]. In dogfish α-crystallin (Figure 2B), 1.01 moles SH/mole were in a slowly reacting class (k =0.0048 min^-1^) and 0.12 moles/mole were in a more rapidly reacting, exposed class (k =0.26 min^-1^). The small amount (0.08 moles/mole) of very rapidly reacting (k = 4 min^-1^) thiol was probably residual DTT which had not been completely removed during dialysis of the protein.

**Figure 3:**
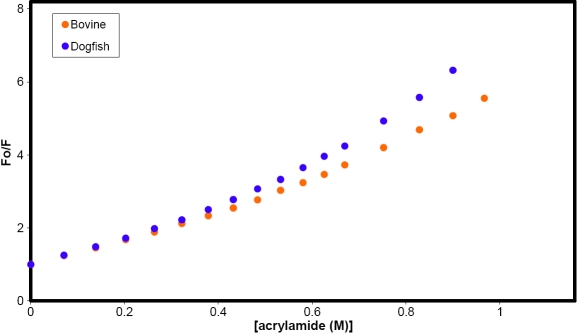
Stern-Volmer plot for the acrylamide quenching of tryptophan fluorescence in bovine and dogfish α-crystallins. Aliquots of 3M acrylamide were added to the proteins (0.05 mg/ml) and the fluorescence emitted at 335nm, after excitation at 295 nm, was measured.

Tryptophan microenvironments were examined by monitoring the quenching of fluorescence using acrylamide. The Stern-Volmer (SV) plots for the two proteins are shown in Figure 3A. The data obtained for bovine α-crystallin are identical to those previously reported [18].

Both proteins yield SV plots with upwards curvature, indicative of static quenching. Correction for this, as described previously [18], reveals that tryptophan fluorescence is more easily quenched in the dogfish protein than in the bovine protein. This can be attributed to the dogfish αB-crystallin polypeptide which contains three tryptophan residues (at positions 9, 49, and 63) and accounts for >80% of the total protein. The αA-crystallin chain contains tryptophan at positions 9 and 154. Comparison with the previously described accessibilities in bovine αB-crystallin [18], which contains tryptophan residues equivalent to those in positions 9 and 63 of the dogfish protein, permitted estimation of the quenching constant (K_dyn_, 2.6 M^-1^) and quantum yield (0.18) for W49 and leads to the conclusion that this residue is in a similar environment to W63 (K_dyn_=2.6; quantum yield=0.18), i.e. more accessible to the solvent and acrylamide than W9 (K_dyn_=2.11: quantum yield=0.14). It was not possible to identify fluorescence originating from W154 in the αA chain because of its low abundance (<6% of total fluorescence).

### Chaperone activity

**Figure 4:**
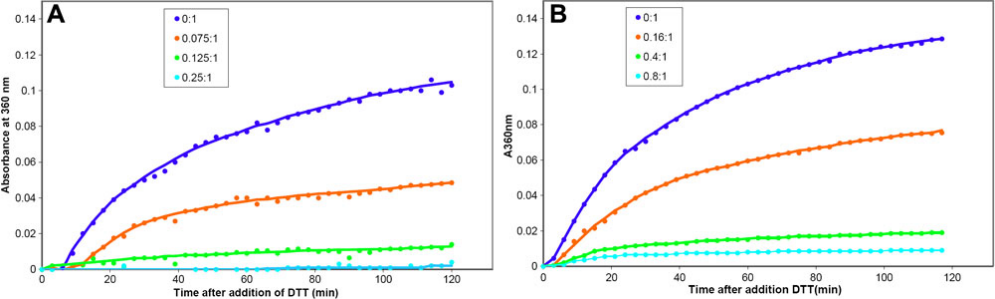
Chaperone activity with insulin reduction. Insulin (0.42 mg/ml) aggregation in the presence and absence of (**A**) dogfish and (**B**) bovine α-crystallins. The proteins were incubated in 50 mM sodium phosphate buffer, 0.03% (w/v) NaN_3_, pH 7.2, at 37 ^°^C. Aggregation of insulin was initiated with 20 mM DTT. The molar ratios of insulin:α-crystallin are indicated.

Two different assay systems, insulin reduction and β_L_-crystallin heat denaturation, were used to compare the chaperone activity of dogfish α-crystallin with that of the bovine protein. Typical data from the insulin reduction assay are presented in Figure 4.

Both proteins were effective at suppressing the aggregation of insulin over a range of concentrations. At a 0.075:1.0 molar ratio of α-crystallin to insulin, dogfish α-crystallin suppressed aggregation by 53% (Figure 4A). By comparison, bovine α-crystallin at a 0.16:1.0 molar ratio only suppressed 41% of the aggregation (Figure 4B). Thus in this reduction assay, dogfish α-crystallin appears to be almost three times more efficient as a chaperone.

**Figure 5:**
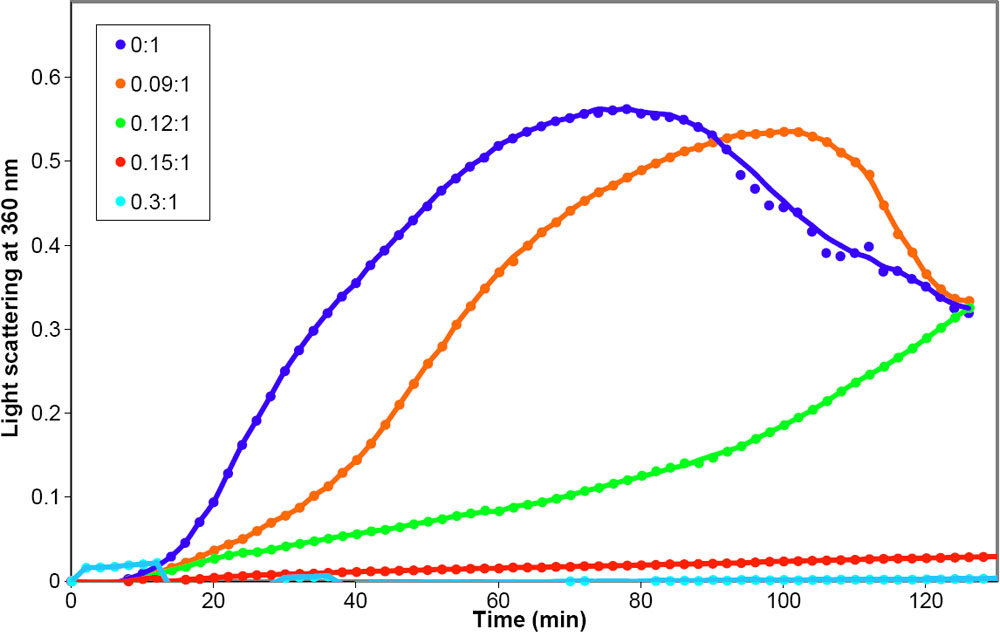
Chaperone activity with heat denatured b-crystallin. β_L_-crystallin (0.3 mg/ml) aggregation in the presence and absence of different concentrations of dogfish α-crystallin. The proteins were incubated in 50 mM sodium phosphate buffer, 0.03% (w/v) NaN_3_, pH 7.2 at 60 ^°^C. The molar ratios of β_L_-crystallin:dogfish α-crystallin are indicated.

Figure 5 demonstrates that increasing concentrations of dogfish α-crystallin were able to reduce the heat-induced precipitation of bovine β_L_-crystallin at 60 °C. A 0.3:1.0 molar ratio of dogfish α-crystallin to β_L_-crystallin suppressed aggregation by 97% and a 0.15:1.0 ratio, by 93%. Under the same conditions, a 0.25:1.0 molar ratio of bovine α-crystallin to β_L_-crystallin completely suppressed the aggregation (data not shown). This suggests that bovine α-crystallin may be a slightly better chaperone than dogfish α-crystallin. When the temperature of the denaturation was increased from 60 to 80 ^°^C, the chaperone activity of bovine α-crystallin increased but that of the dogfish protein was completely abolished, suggesting that the dogfish α-crystallin was less stable than the bovine protein at higher temperatures. This was confirmed by heating dogfish α-crystallin at 80 ^°^C which led to its rapid precipitation. By contrast, dogfish α-crystallin remained soluble for at least 2 h at 60 °C.

### NMR spectroscopy

Two-dimensional (2D) ^1^H-^1^H NMR spectra were acquired on dogfish α-crystallin to determine if the protein contained a flexible COOH-terminal extension as is observed in other sHsps [22,24,25]. Since dogfish α-crystallin exists as large heterogeneous aggregates, it would be expected to give rise to poorly-resolved, broad resonances in the NMR spectrum due to its long tumbling time. However, the one-dimensional (1D) ^1^H NMR spectrum (not shown) exhibited a series of well-resolved resonances superimposed on a broad component, implying that the protein had a region or regions of flexibility present, which may arise from the extreme COOH-terminus. No differences were observed in the 1D ^1^H NMR spectra of before and after 2D data acquisition, indicating that the conformation of the protein was stable for the duration of the NMR experiments (24 h at 25 °C).

**Figure 6:**
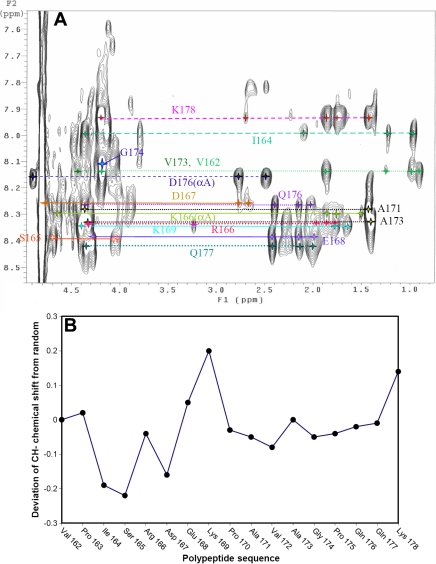
Two-dimensional TOCSY spectrum of dogfish a-crystallin. **A**: The NH to α, β and γ ^1^H proton region of the TOCSY spectrum of dogfish α-crystallin at 25 °C. The assignments are indicated for the αB subunit. **B**: Deviation from random coil chemical shifts [26] for the α-CH ^1^H resonances of the COOH-terminal extension of dogfish α-crystallin.

Intra-residue cross-peaks from the NH protons in the 2D TOCSY spectra, arising from residues in the extreme COOH-terminal region of dogfish αB-crystallin are shown in Figure 6A. Very few cross-peaks from the αA-crystallin subunit were observed in the spectra due to its low concentration relative to the αB-crystallin subunit in dogfish α-crystallin. The identification of the resolved resonances in the spectrum was achieved via the sequential assignment procedure [19] utilizing through-bond, intra-residue TOCSY spectra and through-space, inter-residue connectivities via NOESY spectra. From these spectra, it was apparent that the well-resolved resonances arise from a 17-residue flexible COOH-terminal extension encompassing V162 to K178. The resonances gave rise mainly to sequential NH_i+1_ to α-CH_i_ cross-peaks in the NOESY spectrum which is consistent with the COOH-terminal extension adopting an extended conformation. Proline residues only give cross peaks in the aliphatic region of the spectrum since they have no NH proton. Thus, for the three resolved proline amino acid resonances, sequential assignment was achieved via nOes from the δ-CH_2_ proline resonances to the preceding α-CH amino acid [19].

The α-CH chemical shift of an amino acid in a protein is very sensitive to its local secondary structure [26,27]. A comparison was made between the α-CH chemical shifts determined for resonances in the ^1^H NMR spectrum of dogfish αB-crystallin and those given by amino acids in a random coil environment when followed by either an alanine or a proline residue (Figure 6B). The α-CH ^1^H chemical shifts for dogfish αB-crystallin are within ±0.2 ppm of the random coil values, indicating that they originate from protons that are in a region of the protein that has little, if any, ordered structure and, because the resonances are well resolved in a protein of such size, is highly dynamic and flexible [26].

**Figure 7:**
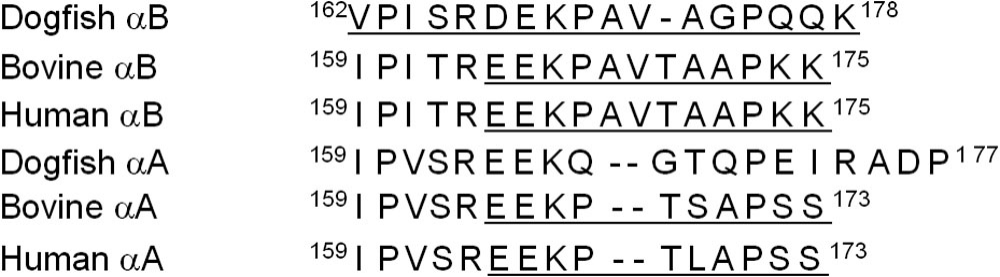
Amino acid sequences in the COOH-terminal region of the A and B polypeptides of dogfish, human and bovine α-crystallins [9]. The NMR-observable flexible COOH-terminal extensions are underlined.

In summary, from Figure 6 and Table 1, it is apparent that the last 17 amino acids of the αB-crystallin subunit (V162-K178) have great conformational flexibility and are highly exposed to the solvent and adopt little ordered conformation. The COOH-terminal sequences of bovine, human and dogfish polypeptides are shown in Figure 7, with the flexible regions indicated.

**Table 1:** ^1^H chemical shifts of the COOH-terminal residues of dogfish αB-crystallin in 10 mM phosphate buffer, pH 6.5, at 25 ^°^C.

**Residue**	**NH**	**Hα**	**Hβ**	**Others**
V162	8.14	4.44	1.86	γCH3, 0.98
P163		4.40	1.88	
I164	8.00	4.36	2.09	γCH2, 1.22; γCH3, 0.96
S165	8.39	4.69	4.06, 4.03	
R166	8.32	4.38	1.85, 1.97	γCH2, 1.75; δCH2, 3.22
D167	8.26	4.80	2.65, 2.75	
E168	8.38	4.30	2.01, 2.14	γCH2, 2.41
K169	8.34	4.40	1.64, 1.78	
P170		4.45	2.28	
A171	8.30	4.37	1.44	
V172	8.14	4.20	1.86	γCH3, 0.90
A173	8.31	4.32	1.39	
G174	8.12	4.18		
P175		4.46	1.92	γCH2.20, 1.98; δCH3.64, 3.66
Q176	8.26	4.36	2.02, 2.14	γCH2, 2.38
Q177	8.42	4.35	1.99, 2.13	γCH2 2.41
K178	7.94	4.18	1.73, 1.85	γCH2 1.41, εCH2 2.69

## Discussion

The spiny dogfish is very old in evolutionary terms, having arisen 420-430 million years ago, about 160 million years before the mammalian line [28]. The bony fish diverged around 30 million years later. Thus, the genes (paralogs) for the dogfish α-crystallins and those which ultimately gave rise to the modern mammalian proteins evolved independently for around 400 million years. This is reflected in the amino acid sequences, which show dogfish αA- and αB-crystallins differ by about 33% from their mammalian counterparts [9,29]. Given the variation in their amino acid sequences, it is possible that dogfish and mammalian proteins would exhibit differences in their structures and functions. Furthermore, the dogfish and mammalian proteins exist and function in very different environments. The ectothermic dogfish is found in temperate waters of 10-18 °C. Its lens is very hard and contains >60% protein. Most mammalian lenses, such as the bovine used in the present work, are relatively soft, with protein concentrations of 30-40% [30] and are maintained at a constant 36-37 °C. The ready availability of dogfish lenses provided an opportunity to determine whether the structure and function of α-crystallins have changed during evolution and/or as a result of adaptation to different environments.

In previous studies on fish α-crystallins, difficulties were encountered in separating α- and β-crystallins [2,11,13]. This was not the case in the present work and may be related to the chromatography medium (S300) and the buffer (PBSA) used, or that the separations were performed at room temperature. Each of these factors might affect the interactions between the crystallins and influence their chromatographic separation. Furthermore, it should be noted that the protein used in this work had been purified by twice rechromatographing the central portion of the peaks. Dogfish α-crystallin was isolated as a smaller aggregate (S_20,w_=14 S;~400kDa in mass) than the bovine protein (~600kDa) but was very similar in size to carp α-crystallin (~410 kDa) [2] and recombinant catfish αB-crystallin (S_20,w_=14.5 S; ~450 kDa) [14]. It is not known what the size of the protein is in the intact lens. However, previous studies have shown that aggregate size has no effect on amino acid microenvironments, immunological reactivity, or chaperone activity in bovine α-crystallins and it is possible to isolate bovine α-crystallins of sizes varying from 160 kDa to >2 MDa [1,31]. Furthermore, construction of heteropolymers from purified bovine subunits yields lower molecular mass aggregates when the proportion of αB-crystallin exceeds 50% [16]. Thus, the observed smaller size of dogfish α-crystallin may reflect its significantly higher αB-crystallin subunit content.

Probing of the microenvironments of cysteine and tryptophan residues revealed similarities between the proteins. Both dogfish polypeptides and the bovine αA chain have a cysteine residue in position 132 (bovine numbering). Dogfish αA-crystallin chains have an additional two cysteines at positions 142 and 143 [29]. Based on the αA/αB-crystallin ratios of 3:1 and 1:5 in bovine and dogfish α-crystallins, respectively, averages of 0.66 and 1.32 SH/mole subunit may be calculated for the two proteins, in good agreement with the observed values of 0.66 and 1.22 SH/mole of subunit as determined from the reaction with DTNB. From the kinetic analysis of the SH reactivities, it can be concluded that Cys-132 is in a buried location in the dogfish subunits in the conserved α-crystallin domain, shielded by the COOH-terminus, as has been previously reported for the bovine αA-crystallin subunit [17]. The two additional cysteines in the dogfish αA-crystallin chain react more rapidly, indicative of groups exposed on the surface of the protein. These residues are near the invariant Pro-145, which marks the end of the α-crystallin domain and the beginning of the largely unstructured COOH-terminus of the protein. Our NMR observations indicate that much of this region is highly exposed to the solvent.

All of the proteins have a tryptophan in position 9. It has been shown previously that this residue is in a shielded position [18]. The tryptophans at positions 49 and 63 of the dogfish αB-crystallin are within the second sequence repeat of the NH_2_-terminal domain, which is responsible for the aggregation of subunits [32]. They are more accessible to quenchers than W9, as is residue 63 in the bovine αB-crystallin polypeptide, but are still well shielded from the solvent.

The similarities between the bovine and dogfish proteins extend to the chaperone activities. Dogfish α-crystallin is also able to prevent the precipitation of reduced insulin and heat-denatured β-crystallins. However, the dogfish protein appeared to be 2-3 times more effective than bovine α-crystallin in preventing the precipitation of reduced insulin at 37 ^°^C. Dogfish α-crystallin was slightly less effective in its ability to suppress precipitation of heat denatured β_L_-crystallin at 60 °C. This may be related to the structural change that α-crystallin undergoes around 45 ^°^C leading to alterations in chaperone ability [33-36] and to the instability of the dogfish protein at elevated temperatures. The variation in the chaperone ability of dogfish and bovine α-crystallin in the insulin reduction assay at 37 ^°^C does not arise from differences in the flexibility of the COOH-terminal flexibility of the two proteins or from alterations in the environment of cysteine and tryptophan residues. Instead, this difference must be ascribed to subtle structural alterations between the two proteins.

It is difficult to compare the chaperone ability of the dogfish protein with that of other ectothermic α-crystallins because of the wide variety of target proteins, stresses and solvent conditions employed in the assays, as well as the endothermic α-crystallins that have been used for comparisons. It has been noted that the chaperone ability of recombinant human αB-crystallin is highly dependent on the solvent conditions and the type of stress [37,38]. As a result of these factors, there is no obvious pattern to the chaperone properties of the fish crystallins, i.e. there are substantial differences between the ectothermic α-crystallins, as well as between their isolated subunits. For example, tuna and toothfish α-crystallins are equally effective in preventing the precipitation of the (same species of) heated γ-crystallins. However, the toothfish protein is about two times more effective than the tuna protein in preventing the precipitation of reduced lysozyme. Differences are also apparent in the activities of the subunits. Zebrafish αB1-crystallin is less stable and a very poor chaperone compared with the zebrafish αA- and the human αB-crystallins. By contrast, catfish αB-crystallin is more stable and is better able to suppress precipitation of heated target proteins than even the endothermic porcine protein [14]. However, there is also a second zebrafish  αB-crystallin subunit (αB_2_), which is about two times better as a chaperone than human αB-crystallin [11]. Dogfish α-crystallin appears to lie in between the two extremes.

It would appear from our various observations, presented in this paper, that the dogfish and bovine α-crystallins have similar subunit structures. This conclusion is supported by previous reports. Puri et al. [39] found that α-crystallins from many vertebrates, e.g. cow, horse, rabbit, rat, and spiny dogfish, are recognized by antibodies raised against bovine α-crystallin. They concluded that the regions of the α-crystallin protein responsible for antibody binding have been conserved and did not change during evolution. This would be consistent with the observation that dogfish α-crystallin polypeptides can form hetero-oligomers with the polypeptides from other species [16]. Leunissen et al. [40] proposed that eye lens crystallins have avoided extreme changes in their charge during evolution. In the lens environment, this could be necessary since changes in the distribution of charged amino acid residues could affect the correct close packing of the crystallins [29].

Our NMR observations revealed that the last 17 amino acids of the B subunit of dogfish α-crystallin (V162-K178) have great conformational flexibility and are highly exposed to the solvent and adopt little ordered conformation (Figure 7). Dogfish αB-crystallin is therefore like the mammalian sHsps (αA- and αB-crystallins, Hsp25 and Hsp20) in having a flexible COOH-terminal extension [24,25]. This region is important in the chaperone action of all mammalian sHsps [41-45]. Removal or alterations of amino acids in this region lead to variations in chaperone activity. Based on sequence alignment, the COOH-terminal extension in dogfish α-crystallin correlates with that observed for human αA- and αB-crystallin. In bovine αB-crystallin, the NMR-observable, flexible extension encompasses the last 12 amino acids whereas it is five amino acids longer in dogfish αB-crystallin and includes the highly conserved IXI/V motif in sHsps (V162, P163, I164 in dogfish αB-crystallin, Figure 7). The X-ray crystal structures of the sHsps, wheat Hsp16.9 and *Methanococcus jannaschii* Hsp16.5 [46], reveal that the IXI/V motif is important in regulating, via subunit-subunit interactions, the oligomeric structure of sHsps.

The COOH-terminal extensions in bovine, dogfish and human αB-crystallins exhibit significant sequence conservation, far more than for the αA-crystallins from these three species, particularly after K169 (Figure 7). The two aspartic/glutamic acid residues, at positions 167 and 168 in dogfish, are entirely conserved in all αA- and αB-crystallins. This is consistent with their importance in the stability and chaperone function of human αB-crystallin and the closely related mammalian sHsp, Hsp25 [45,47].

Thus, the NMR and other observations presented here indicate that dogfish and mammalian α-crystallins adopt a similar structure, which has been maintained over 400 million years of evolution. In particular, a highly flexible COOH-terminal extension is a characteristic feature of heterogeneous sHsps.

We conclude that there has been little or no change in the properties of α-crystallin during the accessible evolutionary period. Given the slow rate of amino acid substitution in the sHsp family [29], perhaps, this is not surprising.
